# Introductory Lecture to the Course of 1877-78 in Atlanta Medical College

**Published:** 1877-10

**Authors:** J. G. Westmoreland

**Affiliations:** Atlanta, Ga.


### INTRODUCTORY LECTURE TO THE COURSE OF 1877-8 IN ATLANTA MEDICAL COLLEGE.

By J. G. WESTMORELAND, M.D., Atlanta, Ga.

*Gentlemen of the Medical Class:*

By the appointment of my confreres it becomes my pleasing duty to welcome you to these halls.

Annually, for about twenty-two years, except, herhaps, three or four years during the war, I have met here the votaries of science, and have always felt a lively interest in their efforts to master the healing art.

To-day, young gentlemen, my anxiety in your behalf is equal to that on any former occasion of this kind, and whenever it becomes my lot to engage your attention, be assured, the teaching shall consist of practical facts, which are to serve you in after life during your professional career, and I feel warranted in promising the same for my colleagues, during the ensuing course of lectures.

It is usual for addresses delivered on occasions like this, to consist principally of the history of medicine and a notice of its prominent founders. Such names as Galen, Bell, Harvey and even that of our ideal father, Æsculapius, afford generally the theme of introductory lectures. It is usual, also, to devote more attention to the literary aspect of such productions, than to the number and arrangement of useful and practical facts. A fascinating display of rhetoric and elocution captivates the hearer who seeks nothing more solid and profitable, and indeed all are pleased with perfection in these respects, however devoid of precepts and principles which are to direct our wandering footsteps in future. Hence the care with which sentences are rounded and classical words selected to adorn public addresses, describing the literary curiosities of past ages.

My purpose, to-day, however, is different. I do not intend to speak of what has been done in medicine, but shall call your attention to the duties of the present and future, and, I hope, in a manner to be understood.

While we look back with pride and gratitude upon the grand discoveries of which members of the profession and the public are happy beneficiaries to-day, we must consider the duties of the hour much more important practically, and feel that they should particularly engage our attention, now.

To you who are preparing for the active duties of our calling, a few general thoughts on the obligations and relations which exist between members of the medical profession and the public, and the importance of correct habits of thought and action, are more appropriate and useful. I have selected, therefore, for my subject on this occasion, *The Medical Profession.*

Few avocations afford less certainty of affluence than ours, and none are more intimately connected with the cause of humanity. Although medical men rarely become rich by the legitimate results of professional labor, they never fail to secure a competency, by the exercise of industry and ordinary frugality. Indeed, opulence is likely to interfere with our regular medical duties, for the attention necessary to preserve a large estate precludes the possibility of professional service to the public. Moreover, the sordid inclinations of him whose heart is fixed on money, are not in unison with the cause of beneficence.

None of the learned professions are intended exclusively for the accumulation of wealth. They are made honorable by their intimate connection with the public interest; and while the "laborer is worthy of his hire," and the honorarium which custom has fixed for their support is amply sufficient to meet all legitimate wants, their ambition is not by any means limited to the acquisition of riches. Proficiency in science brings comfort, joy, happiness to our race, and honor, renown and glory—which money cannot buy—upon the head of those who bestow such blessings.

The world looks upon physicians as the benefactors of mankind, and the misdeeds of charlatans and unprincipled members of the profession only tend to brighten the jeweled wreath on the brow of the truly honorable and upright medical man.

Our obligations to the public are sacred, and when conscientiously fulfilled, serve as inseparable ties, inspiring the hearts of mankind with perpetual admiration. Talk not to me of laws to compel respect from the public and the fulfillment of reciprocal obligations. Human statutes tend rather to weaken that sublime connection wrought by nature, and cemented by philanthropy and science.

The family circle knows no secret that is not confided to the honorable physician. He is the adviser in sickness and trouble, the umpire in domestic dissentions. His is the sacred duty, and to him is awarded the honor of protecting the family, aye, whole communities, from the ravages of epidemics, by the direction which none other can give to public sanitary police.

Notwithstanding this, Georgians must recognize the humiliating fact, that while individuals may appreciate these gratuitous services, the legislative department of this State demands tribute of the self-sacrificing physician, for the poor privilege of serving the public without pay. His charities are rewarded by a tax of ten dollars by the State, and authority for municipal governments to levy fifteen more. We are also insulted in the reason given for this outrage upon our purse, with the statement that protection by the commonwealth in the right to practice our profession justifies the special tax. Insult upon injury! The legal requirement by which the practitioner must give evidence of medical proficiency, serves to protect the public against incompetent physicians, instead of protecting the medical profession.

If, therefore, the people are recipients of benefit from the licensing of physicians, they should of course pay the tax instead of medical men. Not only so, but the discrimination made in the collecting laws of Georgia, against physicians, shows injustice also in the law-making power toward us, and a want of appreciation of the public benefits derived from medical science. These things should not be. A righteous public and an injured profession should see to it that justice be done.

Ours is a mission not less responsible than that intended for the spiritual welfare of mankind. The divine law is complete, and requires only to be made known in its already perfected state, while the science of medicine is imperfect and progressive, requiring thought and judgment in its exercises, for the preservation of life. In order to fill this great mission certain qualities are highly necessary, and the faculty of exercising common sense should have a place at the head of the list. A bombastic display of high sounding technical terms and impracticable theories, betrays a want of solid worth, and makes an unfavorable impression on intelligent people. Occasionally a shallow pate estimates proficiency by unusual expressions, which he does not understand, but remember, gentlemen, that all sensible persons like to understand what you say. Particularly is this necessary in our connection with legal investigations. The statements of medical men made before courts and juries are sometimes indispensable to the ends of justice, and the legal rights of individvals; and in order to the accomplishment of these, the evidence afforded must be understood by the arbiters.

Upon the formation of proper habits, perhaps, more than all else, depends our success in life. The adage, "habit is second nature," should never grow so old as to be lost sight of, nor become so common as to be disregarded. Good and bad practices are alike somewhat uncontrollable when indulged in for a number of years. Excepting immoral habits, none are more damaging to future prospect of success than indolence—*laziness*. This includes neglect of studies, a want of promptness and punctuality; in a word, disinclination to perform our duties generally.

It is true that some are constitutionally opposed to work, and require a greater amount of effort than others; but by the proper exercise of WILL, all may attain to reasonable success. The divine injunction to "mortify the deeds of the flesh," must, in order to success, be practiced in every pursuit of life. We must often act in opposition to our natural inclinations, being directed by judgment rather than taste. Persistance in this course of self-denial forms correct habits, and so changes our natural inclinations as to make duty pleasant and life's journey comparatively easy.

Our intercourse with the public requires, above all things, punctuality. Nothing is more conducive to professional confidence, than systematic engagements and their prompt fulfillment. Indulge not the thought of postponing the time of engagements, however unimportant they may be. It is by neglect of this that a habit is formed embarrassing to our prospects through life, without years of effort to overcome it. Particularly in your pupilage, young gentlemen, let system and time direct your movements, and when you grow older you will not likely depart from these good rules. One minute's failure to be on time rasps the conscience of the truly punctual. Let not the charge of fastidiousness, by your loose associates, deter you from the practice of this important virtue.

You who have not already done so, should now form your habits for life—habits of systematic industry and promptness. Make it a rule of life not to defer till to-morrow what you know should be done to-day. Much of the trouble and hardships which otherwise come upon us, may be thus avoided; and success often depends upon the observance of this simple precept.

Excuse me for thus urging you to cultivate these traits of character, which may be considered of minor importance by some, but which really lie at the foundation of success.

A great misfortune, not so much to the profession of medicine as to the public, is found in the existence of charlatanry and humbuggery, as practiced by the various sects of quacks claiming connection with the body medical. These insatiate parasites prey not so much upon the body to which they try to adhere, as upon the public, whom they deceive and rob. Some of this horde are backsliders from honorable medicine, having received the degree, and in open violation of their obligations and sacred professional honor, have prostituted all to the unholy cause of charlatantry. Others "climb up some other way," as "thieves and robbers," assuming before their intended victims the insignia of authority without ever having attained to that distinction by a knowledge of medical science; while others again find success in the sale of nostrums, without any title at all, by the liberal advertisement of bogus miraculous cures. All, however, are styled Doctors by the dupes of their pretended skill. All have a supposed connection with the medical profession, and by this, lower the dignity and respectability of our calling. In this way, and this alone, are we the sufferers.

The cases, of which for a time we are deprived by these pretenders, in the main are made to require longer and more remunerative treatment at our hands, owing to the delay thus had by the use of nostrums. If, therefore, we seek to make money at the cost of human suffering, we should never raise our voice against charlatans and nostrum dealers. No, the victims of advertising schemes are the injured parties, being deluded by an array of certificates, fictitious and otherwise, bearing the names of clergymen and statesmen as ignorant of medicine as themselves, and when advised by their family physician against listening to the insinuating wiles of the quack, the caution is often attributed to selfishness on his part, and therefore disregarded.

Now, gentlemen, in the name and in behalf of these, our citizens present to-day, and in the name of our people generally, I again, in conclusion, bid you welcome—not only to these halls of science, but to the society and hospitalities of the city.

You come amongst us as strangers, but we hope, after your brief sojourn, we will part with you as friends worthy of our confidence and respect. I am happy to say that, unlike some other places in which the study of our profession is prosecuted, medical students in Atlanta are not looked upon as outlaws and vagabonds, but as gentlemen entitled to respect. Like all other classes of society, however, occasionally one of immoral habits so conducts himself as to forfeit the respect of all good people; but it is our experience that the same number of men collected from any other business in life, will show an equal, if not greater, proportion given to vice than is usually found in the classes of this college. I can see no reason why this class before me should prove an exception to the general rule; and should one go astray and prove himself given to loose and indolent habits, the whole class, as is often the case in other cities, will not be put under the ban on account of one having erred, who belongs to the same body and is engaged in the same pursuit.

Yes, gentlemen, you are received cordially into social relations with our people, and with a firm confidence that nothing will occur to disturb these relations during your stay in the city.

As welcome sojourners amongst us, you are cordially invited to participate in religious worship at any of our churches, and in the social amusements we enjoy. As students of our grand old profession, we cheerfully grant you every facility, both public and private, to qualify you for its responsible duties.

